# Within species support for the expensive tissue hypothesis: a negative association between brain size and visceral fat storage in females of the Pacific seaweed pipefish

**DOI:** 10.1002/ece3.1873

**Published:** 2016-01-18

**Authors:** Masahito Tsuboi, Jun Shoji, Atsushi Sogabe, Ingrid Ahnesjö, Niclas Kolm

**Affiliations:** ^1^ Department of Ecology and Genetics/Animal EcologyEvolutionary Biology CentreUppsala UniversityNorbyvägen 18DSE‐75236UppsalaSweden; ^2^ Center for Field Science of the Seto Inland SeaHiroshima University5‐8‐1, Minatomachi725‐0024Takehara CityHiroshimaJapan; ^3^ Department of BiologyFaculty of Agriculture and Life ScienceHirosaki University1‐1, Bunkyo‐cho036‐8560HirosakiAomoriJapan; ^4^ Department of Zoology/EthologyStockholm UniversitySvante Arrhenius väg 18BSE‐10691StockholmSweden

**Keywords:** Brain size evolution, sex‐role reversal, Syngnathidae, the expensive tissue hypothesis, trade‐off

## Abstract

The brain is one of the most energetically expensive organs in the vertebrate body. Consequently, the high cost of brain development and maintenance is predicted to constrain adaptive brain size evolution (the expensive tissue hypothesis, ETH). Here, we test the ETH in a teleost fish with predominant female mating competition (reversed sex roles) and male pregnancy, the pacific seaweed pipefish *Syngnathus schlegeli*. The relative size of the brain and other energetically expensive organs (kidney, liver, heart, gut, visceral fat, and ovary/testis) was compared among three groups: pregnant males, nonpregnant males and egg producing females. Brood size in pregnant males was unrelated to brain size or the size of any other organ, whereas positive relationships were found between ovary size, kidney size, and liver size in females. Moreover, we found that the size of energetically expensive organs (brain, heart, gut, kidney, and liver) as well as the amount of visceral fat did not differ between pregnant and nonpregnant males. However, we found marked differences in relative size of the expensive organs between sexes. Females had larger liver and kidney than males, whereas males stored more visceral fat than females. Furthermore, in females we found a negative correlation between brain size and the amount of visceral fat, whereas in males, a positive trend between brain size and both liver and heart size was found. These results suggest that, while the majority of variation in the size of various expensive organs in this species likely reflects that individuals in good condition can afford to allocate resources to several organs, the cost of the expensive brain was visible in the visceral fat content of females, possibly due to the high costs associated with female egg production.

## Introduction

The brain is one of the most metabolically costly organs in the vertebrate body (Mink et al. 1981). The large amount of energy required to develop and maintain brain tissue should therefore impose constraints on brain size evolution (Striedter 2005), despite cognitive benefits of having a large brain (Jerison 1973; Striedter and Northcutt 2006; Kotrschal et al. 2013, 2015). Currently, our understanding of energetic constraints on brain size evolution is mainly based on phylogenetic comparative studies at the macroevolutionary level. For instance, the original study by Aiello and Wheeler (1995) that proposed a trade‐off between brain and gut size (the expensive tissue hypothesis, ETH) drew conclusion from comparative data of several anthropoid primates and humans. Most studies that followed the original ETH were also conducted at the macroevolutionary level (e.g., Isler and Van Schaik 2009; Weisbecker and Goswami 2010; Navarrete et al. 2011; Iglesias et al. 2015; Tsuboi et al. 2015). These comparative studies overall suggest three key energetic aspects of vertebrates that constrain brain size evolution: (1) investment into energetically expensive organs (ETH, Aiello and Wheeler 1995), (2) the pace of reproduction (the energy trade‐off hypothesis, Isler and Van Schaik 2006), and (3) basal metabolic turnover (the direct metabolic constraints hypothesis, Martin 1981). Comparative studies generally agree with the view that the energetic constraints have major influences on brain size diversification across vertebrates [the expensive brain framework, (Isler and Van Schaik 2009)], even though ambiguities still exist in some taxonomic groups (Pitnick et al. 2006; Lemaitre et al. 2009).

An emerging research field in the evolutionary ecology of brain morphology is the investigation of variation in brain size at within species (microevolutionary) levels (Gonda et al. 2013). In combination with comparative studies at the between species (macroevolutionary) levels (Jerison 1973; Striedter 2005), microevolutionary studies can provide insights into how adaptive, plastic, or neutral variation contribute to overall patterns of trait divergence (e.g., Gonda et al. 2013). Examinations of ETH in a microevolutionary perspective are thus important to improve our understanding of the energetic constraints on vertebrate brain size evolution. The few existing studies of ETH at the within species level have provided mixed results. With an experimental approach employing artificial selection on brain size in the guppy, *Poecilia reticulata*, Kotrschal et al. (2013) demonstrated a reduction in gut size and fecundity following an increase in brain size. Using a wild population of the Omei wood frog *Rana omeimontis* found a negative correlation between brain size and gut size (Jin et al. 2015), a pattern that supports the ETH. However, a similar study with the bluehead wrasse *Thalassoma bifasciatum*, Warren and Iglesias (2012) showed that brain size was not related with variation in testis size, which is one of the potentially expensive tissues that can represent a trade‐off with brain size (Pitnick et al. 2006; but see Lemaitre et al. 2009). Although these studies have provided important microevolutionary insights concerning the ETH, our knowledge of within species patterns related to the ETH is still limited, making it difficult to assess whether the ETH is a general model for brain size evolution both at the micro‐ and macroevolutionary level.

**Figure 1:**
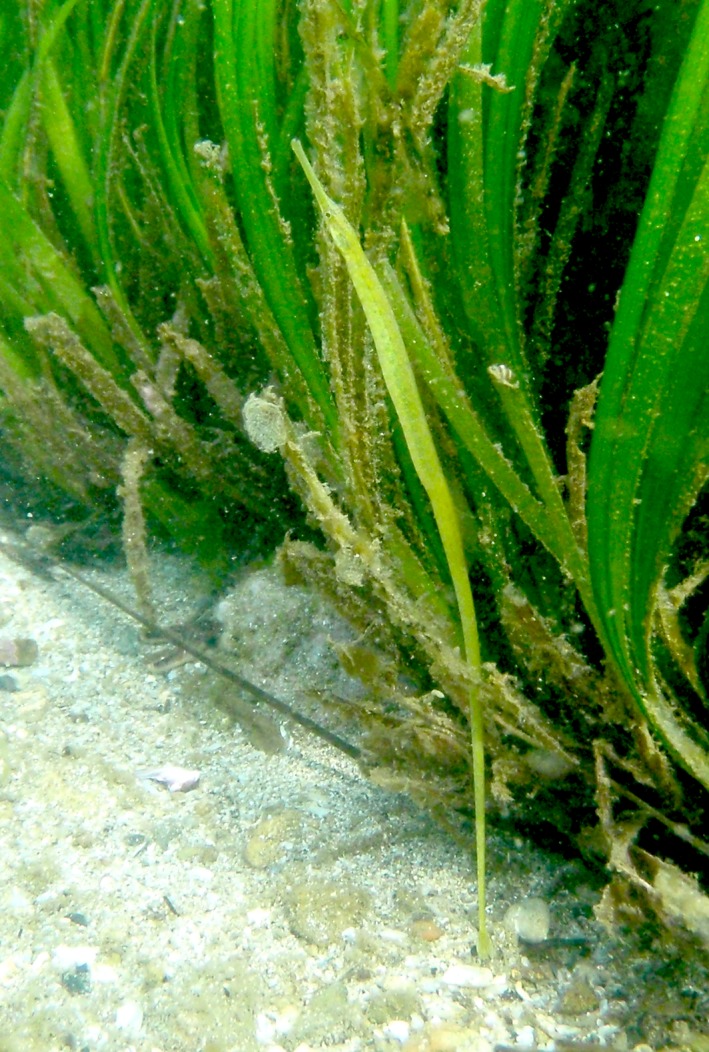
*Syngnathus schlegeli* floating in the eelgrass meadows (*Zostera marina*).

The pacific seaweed pipefish *Syngnathus schlegeli* (Fig. 1) is a member of the teleost family Syngnathidae, characterized by exclusive and often extensive paternal care (Berglund et al. 1986; Wilson et al. 2001; Stolting and Wilson 2007). Males of many syngnathids brood embryos for several weeks depending on the ambient temperature (Foster and Vincent 2004). During this period, brooding males reduce food intake (Svensson 1988; Ahnesjo 1992) while they oxygenate (Goncalves et al. 2015), provide nutrition (Sagebakken et al. 2010; Kvarnemo et al. 2011), and regulate brood pouch osmolarity (Ripley 2009) for the embryos. Like in all other *Syngnathus* species, male *S. schlegeli* provide all postzygotic care of offspring in a brood pouch and brooding takes 14 to 28 days under ambient water temperatures of around 20°C (Watanabe and Watanabe 2001). The mating pattern in this population is most likely characterized by multiple mating by both males and females (i.e., a polygynandrous mating pattern; Sogabe et al. 2012, 2013) and female egg production is asynchronous, thus enabling them to mature eggs continuously and to mate with multiple males in a short time span (Sogabe et al. 2013). The reproductive ecology of this species offers interesting contrasts in reproductive states that can be used to test the ETH at the within species level. The first contrast is between pregnant and nonpregnant males. The second contrast is between nonpregnant males and reproducing females. In accordance with the ETH, we formulate three predictions. First, given that the reproductive investment increases as the number of brooded embryos (i.e., brood size) and produced eggs increase (Ahnesjo 1992), we predict that the size of expensive organs should be negatively correlated with reproductive investment (i.e., brood size in males and ovary size in females). Second, we predict that brooding males and females, which both experience costs associated with reproduction (i.e., egg production by females and embryo brooding by males), have smaller expensive organs than nonbrooding males. Third, we predict that any trade‐off between brain size and other expensive organs will be more likely to exist, and to be more pronounced, in groups with higher energy demands (i.e., brooding males and females) compared to nonbrooding males. By examining the size of the brain and five organs with substantial metabolic costs, gut, heart, liver, kidney, and gonads (Martin and Fuhrman 1955; Aiello and Wheeler 1995) as well as the visceral fat content, a source of energy storage in fish (Reznick and Braun 1987), we thus aim to evaluate the ETH in a microevolutionary perspective.

## Materials and Methods

Sampling of *S. schlegeli* was conducted in the middle of the reproductive season on June 4th 2010 at Otsuchi bay near Nehama beach, Japan (39°20′N, 141°45′E), in shallow eelgrass meadows (*Zostera marina*) using a handheld beach seine. Water temperature during sampling was 16°C. We obtained 20 males that brooded embryos (i.e., brooding males) and all these males brooded embryos in early stages of development (without developed eye spots) as males in later brooding stages were sampled for another study. In addition, 20 males without embryos (i.e., nonbrooding males) and 20 females were sampled. After fixation and preservation in 4% paraformaldehyde in phosphate buffer, we measured the standard length of all specimens (SL, precision = 1.0 mm) using a standard ruler. Following body size measurements, we dissected out the brain and five other organs that have substantial metabolic activity; gut, heart, liver, kidney, gonads (ovary or testis) (Martin and Fuhrman 1955; Aiello and Wheeler 1995) as well as the visceral fat tissue. More specifically, we first removed all the embryos from the brood pouch of brooding males. Then, we opened the body cavity and removed the heart and gastro‐intestinal tract by carefully cutting the posterior end of the esophagus just after the gill and the anterior end of the intestine and reproductive tract. From the removed organs, the swim bladder was detached and heart, gut, liver, and gonads (i.e., testis for males and ovaries for females) were separated. After the visceral fat (attached around the external membrane of the intestine, Henderson and Tocher 1987) was carefully removed, the gut was opened and its content was removed with gentle swabs using a broad‐tipped forceps in a petri dish filled with saline water. The kidney was dissected from the eviscerated body. Finally, the brain was carefully dissected out from the head. Following a few gentle pats on a piece of paper to remove excess fluid, whole brain, heart, gut, kidney, liver, visceral fat tissue, testes, and the whole brood (i.e., all embryos) from brooding males' pouches and ovaries (precision = 0.1 mg) were weighed using MX5 microbalance (Mettler Toledo, Zurich, Switzerland), and this weight was used as the size measurement. All measurements were performed in a period of a single week to minimize potential variation due to differences in fixation time.

All statistical analyses were performed in an R statistical environment Ver. 3.2.1 (R Development Core Team, 2011). First, using a subset of data including either brooding males (*n* = 20) or females (*n* = 20), we assessed the correlation between brood size and expensive organs or visceral fat content. We used a multiple regression with brood size for brooding males and ovary size for females as the response variable and one of log_10_ expensive organ size or log_10_ visceral fat content as a main explanatory variable and log_10_ SL as a covariate. Secondly, we compared the size of the expensive organs (i.e., brain, kidney, liver, heart, gonad, and gut) and visceral fat content between groups with different reproductive state (i.e., nonbrooding male, brooding male or female) using ANCOVA with log_10_ organ weight as a response variable and log_10_ SL as a covariate and reproductive state as an explanatory factor. We excluded interaction terms between log_10_ SL and reproductive state after confirming that they did not have significant effects in any cases. We then assessed the central relationships of the ETH with ANCOVA of log_10_ brain size against each log_10_ organ size (gut, heart, liver, kidney, visceral fat, and testis) as the main explanatory variable, log_10_ SL as a covariate, and reproductive state as a factor. In this model, we introduced an interaction term between reproductive state and organ size, to estimate state‐specific correlations between brain size and organ size in a single model as well as to assess whether the correlation between brain size and other organs/viscera; fat differs between nonbrooding males, brooding males, and females.

## Results

**Figure 2:**
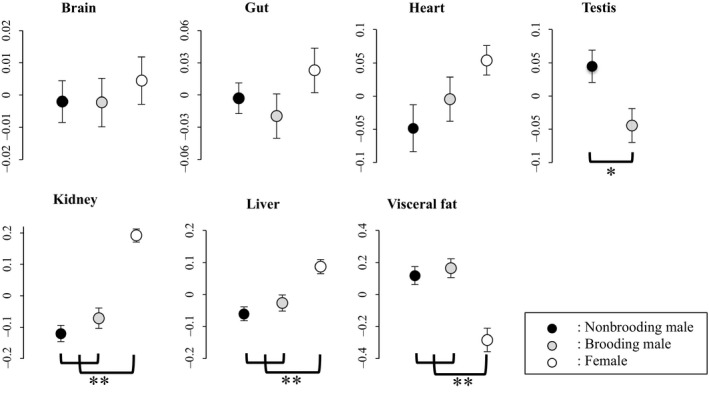
Comparison of relative organ size among nonbrooding males (black circles), brooding males (gray circles), and females (open circles). Size of relative brain, gut, heart, testis, kidney, liver, and visceral fat (i.e., residuals of an ordinary least square regression between log_10_ organ size and log_10_ body length) for each groups with standard errors are presented. Level of significance tested by ANCOVA is represented by * (*P < 0.05, **P < 0.001, see text for details).

**Figure 3:**
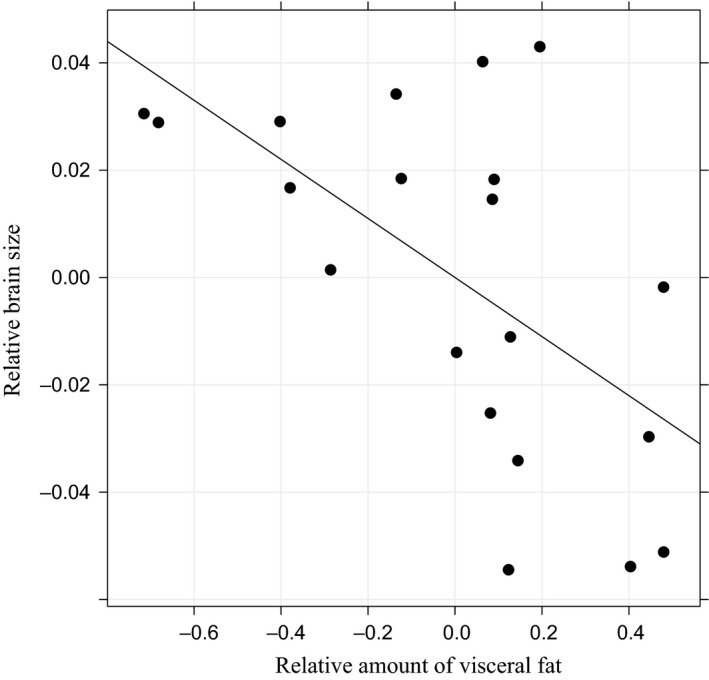
Relationship between brain size and visceral fat storage in female *Syngnathus schlegeli*. Effect of body size was removed from both variables by retaining residuals of an ordinary least square regression against log_10_ body length.

The relationships from the multiple regression analyses between brood size and expensive organs or visceral fat content are summarized in Table 1. Note that, as we included log_10_ body length as a covariate in all models, organ, and visceral fat size are all relative size (i.e., organ size after the variation correlated with body size is removed). We found that brood size (i.e., the total weight of embryos brooded by males) in brooding males was unrelated to any of the expensive tissues investigated in this study (Table 1). In females, however, we found positive significant correlations between ovary size and liver size (Table 1) and kidney size (Table 1). Figure 2 displays the comparison (ANCOVA) of the size of expensive organs in nonbrooding males, brooding males, and females. We found significantly smaller testis size in brooding males compared to nonbrooding males (ANCOVA: reproductive state: *F*_1,37_ = 6.67, mean difference _upper, lower 95% confidence interval (c.i.)_ = 0.09 _0.02, 0.16_, *P *=* *0.014; Fig. 2). We found significant effects of reproductive state on kidney, liver and visceral fat size (ANCOVA: reproductive state: kidney: *F*_2,56_ = 40.32, *P *<* *0.001, liver: *F*_2,56_ = 11.45, *P *<* *0.001, visceral fat: *F*_2,56_ = 15.13, *P *<* *0.001; Fig. 2), but not for brain, gut and heart size (ANCOVA: reproductive state: brain: *F*_2,56_ = 0.28, *P* = 0.75, gut: *F*_2,56_ = 1.28, *P* = 0.29, heart: *F*_2,56_ = 2.79, *P* = 0.07; Fig. 2). Females (F) had larger kidney and liver than brooding (B) and nonbrooding (nB) males (average relative size ± SE: kidney: *F* = 0.19 ± 0.02, *B* = −0.07 ± 0.03, nB = −0.12 ± 0.03, liver: *F* = 0.09 ± 0.02, *B* = −0.03 ± 0.02, nB = −0.06 ± 0.02, Tukey's HSD: kidney: F‐B:, *P *<* *0.001, F‐nB: *P *<* *0.001, liver: F‐B: *P* = 0.003, F‐nB: *P *<* *0.001; Fig. 2) and smaller amounts of visceral fat than brooding and nonbrooding males (average relative size ± SE: visceral fat: *F* = −0.28 ± 0.07, *B* = 0.16 ± 0.06, nB = 0.12 ± 0.06, Tukey's HSD: F‐B: *P *<* *0.001, *F*‐nB: *P *<* *0.001; Fig. 2), while brooding and nonbrooding males did not differ from each other (Tukey's HSD, nB‐B: kidney: *P* = 0.41, liver: *P* = 0.56, visceral fat: *P* = 0.86; Fig. 2). Our assessment of the correlations between brain size and other expensive organs are summarized in Table 2. We found a nonsignificant trend (*P* = 0.053) for a positive correlation between brain size and liver size in nonbrooding males and a negative correlation between brain size and visceral fat content in females (*P* = 0.008, Fig. 3). None of the relationships differed between brooding and nonbrooding males (Table 2) but the correlation between brain size and visceral fat deposit was significantly different between females and nonbrooding males as well as brooding males (Table 2).

**Table 1:** Summary of the associations of brood size and ovary size with other organ sizes and weight of visceral fat. The correlation coefficient (*r*) ± standard error, *t*‐value, degrees of freedom, and *P*‐value are presented. Significant results (*p* < 0.05) are presented with a bold font. Note that, in all analyses, log_10_ body length was included to control for the effect of allometry

Trait	*r* ± SE	*t*_17_	*P*
Brain			
Brood	−0.18 ± 0.22	−0.81	0.42
Ovary	0.09 ± 0.18	0.48	0.64
Gut			
Brood	0.14 ± 0.23	0.61	0.55
Ovary	0.045 ± 0.176	0.26	0.80
Liver			
Brood	0.02 ± 0.25	0.08	0.94
Ovary	0.50 ± 0.20	2.52	**0.022**
Heart			
Brood	−0.01 ± 0.25	−0.06	0.95
Ovary	0.08 ± 0.24	0.32	0.75
Kidney			
Brood	−0.07 ± 0.24	−0.29	0.77
Ovary	0.42 ± 0.17	2.53	**0.021**
Fat			
Brood	−0.22 ± 0.25	−0.88	0.39
Ovary	0.17 ± 0.24	0.73	0.47
Testis			
Brood	0.29 ± 0.23	1.25	0.23

**Table 2:** Summary of ANCOVA testing correlations between brain size and the size of other organs and comparing them among three reproductive states (nonbrooding, brooding males, females). The correlation coefficient (*r*) ± standard error, *t*‐value, degrees of freedom, and *P*‐value are presented. Significant results (*p* < 0.05) are presented with a bold font. Note that, in all analyses, log_10_ body length was included to control for the effect of allometry

Trait	Gut			Liver			Heart		
	*r* ± SE	*t*_53_	*P*	*r* ± SE	*t*_53_	*P*	*r* ± SE	*t*_53_	*P*
Nonbrooding male	0.17 ± 0.26	0.66	0.51	0.43 ± 0.22	1.98	0.053	0.24 ± 0.17	1.43	0.16
Brooding male	0.001 ± 0.17	0.007	0.99	0.25 ± 0.20	1.21	0.23	0.27 ± 0.17	1.52	0.14
Female	0.05 ± 0.18	0.29	0.77	−0.11 ± 0.24	−0.48	0.64	−0.08 ± 0.27	−0.28	0.30
Brain × Rep. state _BM‐F_	–	−0.21	0.84	–	1.17	0.25	–	1.07	0.29
Brain × Rep. state _NBM‐F_	–	0.38	0.70	–	1.72	0.09	–	1.00	0.32
Brain × Rep. state _BM‐NBM_	–	−0.56	0.58	–	−0.61	0.54	–	0.11	0.92

	Kidney			Visceral fat			Testis		
Trait	*r* ± SE	*t*_53_	*P*	*r* ± SE	*t*_53_	*P*	*r* ± SE	*t*_53_	*P*
Nonbrooding male	0.21 ± 0.28	0.75	0.46	0.33 ± 0.23	1.41	0.17	0.05 ± 0.07	0.71	0.48
Brooding male	0.04 ± 0.26	0.17	0.87	0.24 ± 0.23	1.00	0.32	0.02 ± 0.06	0.28	0.78
Female	0.009 ± 0.37	0.02	0.98	−0.48 ± 0.18	−2.73	**0.008**	–	–	–
Brain × Rep. state _BM‐F_	–	0.08	0.94	–	2.45	**0.02**	–	–	–
Brain × Rep. state _NBM‐F_	–	0.47	0.64	–	2.79	**0.007**	–	–	–
Brain × Rep. state _BM‐NBM_	–	−0.46	0.65	–	−0.28	0.78	–	0.33	0.74

## Discussion

In the present study, we tested several aspects of the ETH at the within species level. Our data provided three major findings. First, male pregnancy was not related to reductions in any expensive tissue, except for testis mass. Second, we found a marked sexual dimorphism in the size of several expensive organs. For instance, females had relatively heavier liver and kidney compared to males. Third, we found mixed support for ETH. Relative brain size was negatively correlated with weight of visceral fat only in females, whereas relative brain size showed a positive trend with liver size in nonbrooding males. In addition, in females, the size of ovaries correlated positively with liver and kidney size. Below, we discuss these findings in light of the particular reproductive biology of pipefishes and the ETH.

### Costs of male pregnancy

In our comparisons between brooding and nonbrooding males, we found that testis size was significantly reduced within brooding males. Given that fertilization in *S. schlegeli* occurs within the brooding structure after egg transfer from the female (Watanabe et al. 2000), this result is most likely a consequence of brooding males having spent their sperm at mating, when receiving the eggs into their pouch. Contrary to our prediction, we did not find any reduction in other expensive organs in brooding males compared to nonbrooding males. Moreover, we found no relationship between the brood size in males and any other organ size. Overall, our results indicate that the cost of male brooding was limited to sperm reduction with no measureable effect on any of the other metabolically expensive organs or visceral fat content. However, our sample of brooding males was collected shortly after receiving eggs from the females and this bias in our dataset may have masked reductions in expensive organs that may appear in males in later stages of pregnancy. Previous studies of Syngnathidae and their reproductive biology have indicated that the physiological costs of male pregnancy become more evident in the later stage of the pregnancy (Goncalves et al. 2015; Paczolt and Jones 2015). Therefore, the lack of support for any costs of paternal care in light of investment into metabolically expensive organs should be taken with caution. Future studies that include males at different stages of brooding should provide further insights regarding the costs of male pregnancy in this species.

### Sexual dimorphism in organ size

We found marked sexual differences in the size of expensive organs in *S. schlegeli*. Females had a larger heart, kidney and liver than males, a pattern that parallels results reported in another population of *S. schlegeli* (Sogabe et al. 2012). Conversely, females stored significantly less visceral fat than males and the amount of visceral fat in the females was limited or almost absent. Furthermore, we found positive associations between female ovary size and liver and kidney size. The underlying mechanisms producing these results are likely to be associated with a female's egg production. A greater capacity for physiological activities is generally reflected in a greater size of organs responsible for energy turnover (Daan et al. 1990; Peterson et al. 1990). In *S. schlegeli*, females have the ability to continuously mature oocytes and egg production is asynchronous (Sogabe et al. 2013), like in many other polygamously mating pipefishes (Sogabe and Ahnesjo 2011). This continuous and rapid egg production should increase the synthesis of vitellogenins in the liver (Henderson and Tocher 1987; Tyler and Sumpter 1996; Lubzens et al. 2010), and the associated increase in these activities could benefit from an increased capacity of osmoregulation by larger kidneys. Therefore, elevated physiological activities for continuous egg production may have led female *S. schlegeli* to invest more into the liver and kidney than males. This is further strengthened by the found positive relationships between ovary size and liver/kidney size. However, these organs are also metabolically expensive to maintain (Martin and Fuhrman 1955; Aiello and Wheeler 1995). Consequently, females are likely to spend substantially more of their fat deposits than males, presumably for elevated running and maintenance cost of metabolically active organs. Alternatively, females may allocate acquired resources directly into egg production or other metabolically active organs rather than into fat deposits. Separately or in concert, these processes could have resulted in females having, on average, only 43% of the amount of visceral fat content in comparison to males. Overall, we interpret our results such that the found sexual dimorphism in organ size of *S. schlegeli* is most likely a consequence of the cost of female reproduction.

### The expensive tissue hypothesis

It is widely recognized that the existence of trade‐offs are difficult to demonstrate (Agrawal et al. 2010). The key issue is that, when the variation in resource acquisition is larger than the variation in resource allocation, trade‐offs are masked by strong variation between individuals in resource acquisition (Van Noordwijk and De Jong 1986). Along this line of argument, our detected trend for a positive correlation between brain size and liver size in male *S. schlegeli* as well as the positive relationships between ovary size and liver and kidney size in females are likely to be a reflection of large variation in energy acquisition among individuals in our study. Overall, our results here indicate that individuals in better energetic condition can afford to allocate resources into multiple organs, of high energetic costs, while individuals in worse condition may allocate less resources overall into these organs (e.g., the big car ‐ big house paradox, Van Noordwijk and De Jong 1986). Indeed, Paczolt and Jones (2015) demonstrated that pregnant males of the Gulf pipefish (*Syngnathus scovelli)* in a high food level treatment were able to allocate resources both to brooding and growth, whereas pregnant males in a low food level treatment maintained investment in the current brood and sacrificed somatic growth (Paczolt and Jones (2015). Moreover, positive correlations among relative organ sizes have also been found previously in fishes (Odell et al. 2003; Norin and Malte 2012), amphibians (Jin et al. 2015), and mammals (Chappell et al. 2007). Together, these previous studies and our data indicate that a larger variation in energy acquisition than in energy allocation might be common at within species scales (Glazier 1999). However, our found positive correlations between expensive organs do not necessarily exclude the existence of underlying energetic constraints (Roff 1992; Stearns 1992).

Interestingly, we found a female‐specific negative association between brain size and visceral fat content. As discussed earlier, female pipefishes sacrifice their visceral fat storage for egg production while maintaining larger metabolically costly organs than males. This may have led females to face strong competition over the allocation of resources, both in terms of immediate energy intake from feeding and decomposition of visceral fat storage, between maintenance of brain tissue and ovary development. Importantly, sexual selection is most likely stronger in females than in males in this species (Watanabe et al. 2000). Given that courtship and intrasexual competition are cognitively challenging (Boogert et al. 2011), females of *S. schlegeli* may be under stronger selection to have larger brains than males, on top of the cost associated with rapid and continuous egg production (Sogabe et al. 2013). Our recent demonstration of female‐biased brain size dimorphism across Syngnathidae further strengthens this view (M. Tsuboi, A.C.O Lim, O.L. Ooi, M.Y. Yip, V.C. Chong, I. Ahnesjö, and N. Kolm, unpubl. ms.). The female‐specific negative association between brain size and fat storage supports a previous comparative study in mammals (Navarrete et al. 2011). This may imply the existence of a direct energetic connection between brain maintenance and fat storage. However, following the study of Navarrete et al. (2011), the metabolic connection between fat and brain was challenged by Speijer (2012). The central argument in this critique was that brain tissue generally do not use fatty acid breakdown for energy generation, presumably due to high oxygen radical formation (Speijer 2011). However, brain tissue can use fat as energy source through conversion to ketone bodies such as acetoacetate and *β*‐hydroxybutyrate in the liver (Henderson and Tocher 1987; Soengas and Aldegunde 2002; Speijer 2012). This process is most important during energetically active periods and situations that are presumably common for females of *S. schlegeli* during the reproductive season. Hence, we propose that a trade‐off between brain size and fat storage is possible and detectable at the within species level, at least in our studied species and during highly energy‐demanding activities such as egg production, intrasexual competition, and courtship. In summary, our results support, together with other recent findings (Kotrschal et al. 2013; Jin et al. 2015), that energetic trade‐offs associated with expensive brain tissue may exist at within species level in a similar manner as proposed by macroevolutionary studies (Aiello and Wheeler 1995; Isler and Van Schaik 2009; Tsuboi et al. 2015).

To conclude, our within species investigation of ETH suggests that the energetic cost of the brain may lead to a trade‐off with fat storage as suggested in a previous study at the between species level (Navarrete et al. 2011). Our results indicate that patterns of covariation at the microevolutionary level could be mirrored against patterns at the macroevolutionary level to elucidate general validity of hypotheses in evolutionary ecology. However, our study also highlight that attempts to link micro‐ and macroevolutionary patterns should be performed with careful attention to the substantial differences in the levels of trait variation at different evolutionary scales.

## Conflict of Interest

The authors have no conflict of interest to declare.

## Data Accessibility

Data available from the Dryad Digital Repository: http://dx.doi.org/10.5061/dryad.t25d2
